# Nature of spiral state and absence of electric polarisation in Sr-doped YBaCuFeO_5_ revealed by first-principle study

**DOI:** 10.1038/s41598-018-20774-7

**Published:** 2018-02-05

**Authors:** Dibyendu Dey, S. Nandy, T. Maitra, C. S. Yadav, A. Taraphder

**Affiliations:** 1 Department of Physics, Indian Institute of Technology Kharagpur, Kharagpur, 721302 India; 2 Department of Physics, Indian Institute of Technology Roorkee, Roorkee, 247667 India; 3 School of Basic Sciences, Indian Institute of Technology Mandi, Himachal Pradesh, 175001 India; 4 Centre for Theoretical Studies and Centre for Nanoscience and Nanotechnology, Indian Institute of Technology Kharagpur, Kharagpur, 721302 India

## Abstract

Experimental results on YBaCuFeO_5_, in its incommensurate magnetic phase, appear to disagree on its ferroelectric response. Ambiguity exists on the nature of the spiral magnetic state too. Using first-principles density functional theory (DFT) calculations for the parent compound within LSDA + U + SO approximation, we reveal the nature of spiral state. The helical spiral is found to be more stable below the transition temperature as spins prefer to lie in ab plane. Dzyaloshinskii-Moriya (DM) interaction turns out to be negligibly small and the spin current mechanism is not valid in the helical spiral state, ruling out an electric polarisation from either. These results are in very good agreement with the recent, high quality, single-crystal data. We also investigate the magnetic transition in YBa_1−x_Sr_x_CuFeO_5_ for the entire range (0 ≤ *x* ≤ 1) of doping. The exchange interactions are estimated as a function of doping and a quantum Monte Carlo (QMC) calculation on an effective spin Hamiltonian shows that the paramagnetic to commensurate phase transition temperature increases with doping till *x* = 0.5 and decreases beyond. These observations are consistent with experimental findings.

## Introduction

Type-II multiferroic materials, where ferroelectricity is induced by the spiral magnetic order^1–4^, are expected to have various applications in magnetoelectric sensors, magnetic memory devices, etc. A prime objective is to design materials with high transition temperatures (T_*N*_) for the spiral magnetic state. In systems which are not geometrically frustrated, the spiral state results from competing nearest-neighbour (J_*NN*_) and next-nearest-neighbour exchange (J_*NNN*_) interactions^5^. High values for such interactions are therefore desired for raising the T_*N*_. However, in transition-metal oxides, such interactions are generally weak^6^ and T_*N*_ for the spiral states are low^7,8^.

There is, however, an exception where magnetism-driven ferroelectricity is observed close to room temperature via a stable spiral state: cupric oxide (CuO)^9,10^, has a spiral phase above 200 K, in a narrow window between 213 K to 230 K^9,11^ where a finite electric polarisation is also observed. The monoclinic symmetry with frustrating magnetic interactions and competing NN and NNN exchange interactions stabilize the magnetic spiral in this system around room temperature. The oxygen-deficient layered perovskite YBaCuFeO_5_ (YBCFO) was later reported to display multiferroism at unexpectedly high temperature^12^. Large electric polarisation associated with the spiral phase was reported in ceramic samples of YBCFO^12,13^ within a wide temperature range (almost ten times that of CuO) at zero magnetic field. However, the direction of electric polarisation in YBCFO remained a matter of debate. Kundys *et al*.^12^ proposed that the polarisation vector lies along the c axis due to the formation of dipole moments within the bipyramid, while a later report^13^ suggests cycloidal nature of the spiral, tilted along c axis, favoring electric polarisation perpendicular to z direction.

A very recent experiment by Lai, *et al*.^14^ on single-crystal samples of YBCFO, however, does not find any signature of the electric polarisation. Careful measurements of magnetization, dielectric constant and neutron diffraction on a high quality single crystal show two antiferromagnetic transitions at 475 K and 175 K and a giant dielectric constant, characteristic of a dielectric relaxation at 175 K. Their magnetic data agree well with the previous magnetic measurements and confirm the presence of a spiral order. They suggest that the spins are rotated in the ab plane and form a magnetic spiral without any tilt along c direction.

**Figure 1:**
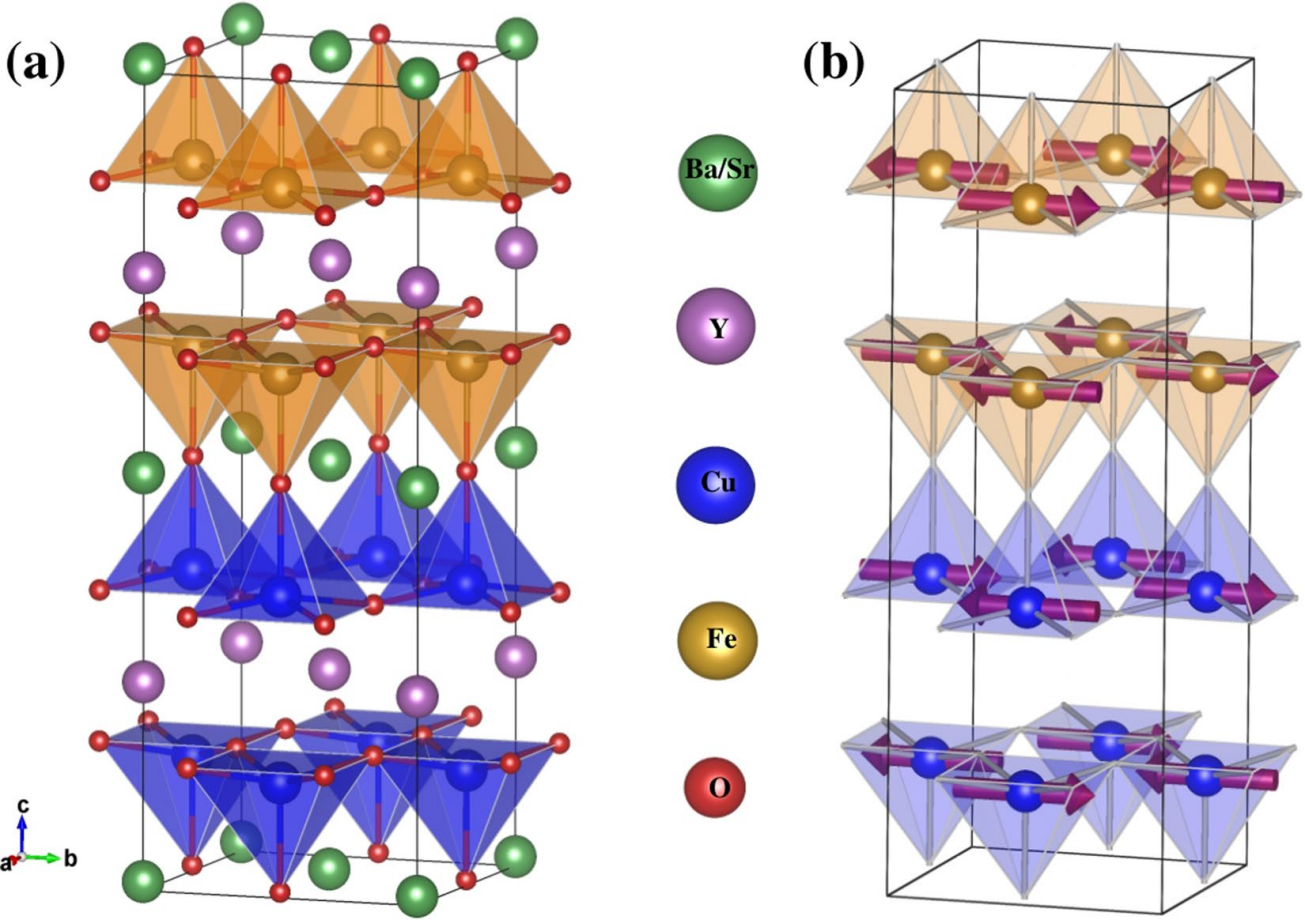
(**a**) $\sqrt{2}\times \sqrt{2}\times 2$ Supercell of YBa_1−*x*_Sr_*x*_CuFeO_5_ showing FeO_5_ and CuO_5_ square pyramids in golden and blue colours respectively. (**b**) Experimentally observed magnetic structure of commensurate phase^14^.

YBCFO crystallizes into a tetragonal structure with *P*4*mm* symmetry^15^. Experimentally it is not possible to assign any ordering of Fe/Cu ions due to their similar ionic radii. However, first-principles density functional theory calculations show that the bipyramidal layers are preferentially occupied by ferromagnetically coupled Fe-Cu pairs^13^ (Fig. 1) within the experimentally observed commensurate magnetic structure. YBCFO undergoes two magnetic transitions^14,16–19^ higher temperature paramagnetic (PM) to commensurate (CM) antiferromagnetic phase transition occurs at *T*_*N*1_ with the magnetic propagation vector *k*_*cm*_ = (1/2, 1/2, 1/2). An incommensurate (ICM) magnetic order sets in at a lower temperature *T*_*N*2_. The neutron powder diffraction (NPD) data shows magnetic propagation vector *k*_*icm*_ = (1/2, 1/2, 1/2 ± *q*) in the ICM phase^14,15^. However, ambiguity remains in the recent experiments^12–14^ the connection between ferroelectric properties and the ICM phase and the nature of magnetic spiral in that regime are still open and unresolved issues.

Controlled introduction of chemical disorder into YBCFO during sample preparation, in the form of Fe-Fe or Cu-Cu impurity bonds, is found to enhance the stability of the magnetic spiral state further^15^, giving rise to a large increase of T_*N*2_ beyond room temperature, as high as 310 K. However, the presence of electric polarization in the sample could not be confirmed in this report. Subsequently, Monte-Carlo simulations have shown that these impurity-bonds introduce a large out of plane antiferromagnetic exchange interaction which stabilizes the cycloidal spiral state that couples to the electric polarization^20,21^. Application of pressure or doping isovalent ions at A sites are believed to have similar effects as chemical disorder described above. Indeed, the enhancement of T_*N*2_ by doping isovalent Sr at Ba sites has been observed in a very recent measurement^22^.

We shed light on some of these issues from first-principles calculations. In particular, we address the following: (i) the spin orientation in the ab plane, (ii) the nature of extant magnetic order - helical spiral or cycloidal, (iii) whether Dzyaloshinskii-Moriya (DM) interaction is present and if there is a possible route to electric polarisation in this system. We compare our results with recent single-crystal data and find good agreement. We discuss earlier data on polycrystalline samples as well and suspect that the apparent discrepancy between different experimental data is likely to be an artifact of measurements on single-crystal versus polycrystal samples. Our theoretical results agree well with the single crystal data.

We investigate the parent YBCFO and its doped variants using first-principles DFT calculations. The paper is structured as follows. First, we discuss the crystal and magnetic structure of both parent and doped compounds in detail followed by the calculation of magnetic exchange interactions in the CM phase to find the origin of the spiral phase. Next we study the effect of spin-orbit coupling (SOC) and calculate the anti-symmetric DM interaction parameter to identify the nature of the spin-spiral state in YBCFO. Finally, we study the effect of A site doping, and follow it by a QMC calculation of an effective spin model whose exchange interactions are derived from the DFT calculations. It would be useful to know how the exchange interactions evolve with doping and how they compete with each other in stabilizing or destabilizing the commensurate and incommensurate (spiral) magnetic phases. We also compare our results with existing experimental data. Finally we give a brief summary and outlook.

## Results

### Crystal and Magnetic Structure

YBa_1−*x*_Sr_*x*_CuFeO_5_ has the layered perovskite structure with a noncentrosymmetric *P*4*mm* space group^22^ for the entire range of doping as shown in Fig. 1(a). The experimental lattice constants of the parent and doped compounds are listed in Table 1. In the tetragonal structure, two square pyramids FeO_5_ and CuO_5_ are connected via apical Oxygen forming a layer of bipyramids. Ba^2+^/Sr^2+^ ions go into the interstitial positions in between the two square pyramidal layers. These bipyramidal layers (or bilayers) are separated by a layer of Y^3+^ ions.

**Table 1:** Experimental lattice constants of YBa_1−*x*_Sr_*x*_CuFeO_5_^22,30,31^.

x	a (Å)	b (Å)	c (Å)
0.00	3.8711	3.8711	7.6629
0.25	3.8603	3.8603	7.6595
0.50	3.8506	3.8506	7.6517
0.75	3.8408	3.8408	7.6342
1.00	3.8317	3.8317	7.6076

In our *ab*-*initio* calculations a supercell with ${{\rm{a}}}_{s}=\sqrt{2}a$ and c_*s*_ = 2c (a and c are the crystallographic unit cell parameters) is considered which contains four formula units as depicted in Fig. 1(a). This construction of the supercell was required to incorporate the CM magnetic structure shown in Fig. 1(b). We have checked the converged energies with larger supercells as well. The above supercell produces accurate results in energies to within the third decimal place (in eV) in comparison with bigger ones, e.g., with 2 × 2 × 2, and $\sqrt{2}\times \sqrt{2}\times 4$. In this magnetic phase, the bipyramidal units connected via apical Oxygen are preferentially occupied by Cu-Fe pairs and the interaction within these Cu-Fe dimers is ferromagnetic (FM). Whereas Cu-Cu and Fe-Fe pairs separated by Y layers are antiferromagnetically coupled. Furthermore, all the nearest-neighbour (NN) interactions within the ab-plane are AFM in nature.

At lower temperatures (below T_*N*2_) a spiral magnetic state is formed in which non-collinear spin arrangements appear within the bipyramidal layer (Cu-Fe pairs)^14^. However, inter-bipyramidal exchange (Cu-Cu and Fe-Fe) interactions remain AFM in the ICM phase.

### Magnetic Exchange Interactions

From our DFT total energy calculations we observe that the parent compound YBCFO, in its commensurate magnetic phase, has the lowest energy when Fe^3+^ and Cu^2+^ ions are preferentially ordered in each bipyramid (Fig. 1(a)), in agreement with earlier reports^13^. Hence we considered this structure for further calculations as discussed below. The nearest (J_*NN*_) and next nearest neighbour (J_*NNN*_) exchange interactions were estimated by mapping the total energy difference between FM and AFM configurations for a pair of spins to the classical Heisenberg Hamiltonian which can be written as

1 $$H=\frac{1}{2}\sum _{ij}{J}_{ij}({{\bf{S}}}_{i}\cdot {{\bf{S}}}_{j})$$

Calculated J values are listed in Table 2 for parent as well as for doped compounds. For the parent compound, the J-values are comparable to the previously reported values^13^. Here *J*_*p*1_ and *J*_*p*2_ are the in-plane Cu-Cu and Fe-Fe NN exchange couplings respectively which are found to be AFM in nature. We also observe that the strongest exchange interaction is in the ab-plane (*J*_*p*1_). The out-of-plane interactions along c (*J*_1_, *J*_2_, *J*_3_ and *J*_4_) are much weaker (almost one order of magnitude less) compared to the in-plane *J*_*p*1_.

**Table 2:** NN and NNN exchange interaction strengths in YBa_1−*x*_Sr_*x*_CuFeO_5_ in meV.

x	J_1_	J_2_	J_3_	J_4_	J_*p*1_	J_*p*2_	J_*NNN*_
0.00	−2.016	−2.016	14.01	2.86	149.5	8.78	−0.069
0.25	−2.383	−1.783	13.52	2.68	150.5	8.95	−0.087
0.50	−2.766	−1.646	13.10	2.59	155.5	9.15	−0.117
0.75	−2.511	−1.940	12.59	2.40	146.7	9.27	−0.095
1.00	−2.325	−2.325	12.27	2.31	147.6	9.41	−0.087

To obtain insight into the origin of ICM phase, we studied the effect of next-nearest-neighbor (J_*NNN*_) exchange interactions along the c direction on the magnetic ordering. One possible way to create magnetic frustration in the unfrustrated CM phase is to have a strong ferromagnetic J_*NNN*_. However, our first-principles estimation of J_*NNN*_ shows that this interaction (when ferromagnetic) is too small (Table 2) to cause enough frustration in destabilizing the CM phase in favour of an ICM one. The NNN interaction strengths for other structures with different Fe^3+^/Cu^2+^ ordering are also found to be either too weak or of wrong sign to stabilize a spiral spin state. In the following section we explore the other possible scenarios for the formation of a spiral state in this system.

### Effect of Spin-Orbit Coupling

In order to determine the spin easy-axis or easy-plane anisotropy of the CM phase in YBCFO, we consider spin-orbit (SO) coupling in our calculations within LSDA + U + SO approach. As noted above, there exists a disagreement on the directions of spin moments in the CM phase of YBCFO in different experiments and it is useful therefore to calculate it here. Comparing total energies for three configurations with spin moments directed along x, y and z directions in the CM phase, we find that the spin moments prefer to lie in the xy-plane rather than along z-direction. The energy difference (*E*_*xy*_ − *E*_*z*_) between them is ≈−3 meV. The single-crystal measurements on YBCFO^14^ agree with our observation: the moments are indeed aligned in the ab-plane.

Moving on to the issue of magnetic spiral state-induced ferroelectricity in the ICM phase, we explore a plausible microscopic mechanism^23,24^ which involves antisymmetric DM interaction^25,26^. This interaction is a relativistic correction to the usual superexchange and is given by the following Hamiltonian

2 $${H}_{DM}=\sum _{{i}j}{{\bf{D}}}_{ij}\cdot [{{\bf{S}}}_{i}\times {{\bf{S}}}_{j}]$$

The strength of the interaction is proportional to the SOC constant. The DM interaction favours non-collinear spin ordering in perovskite manganites^24^. It also transforms the collinear state into a magnetic spiral in ferroelectric materials^27,28^.

**Figure 2:**
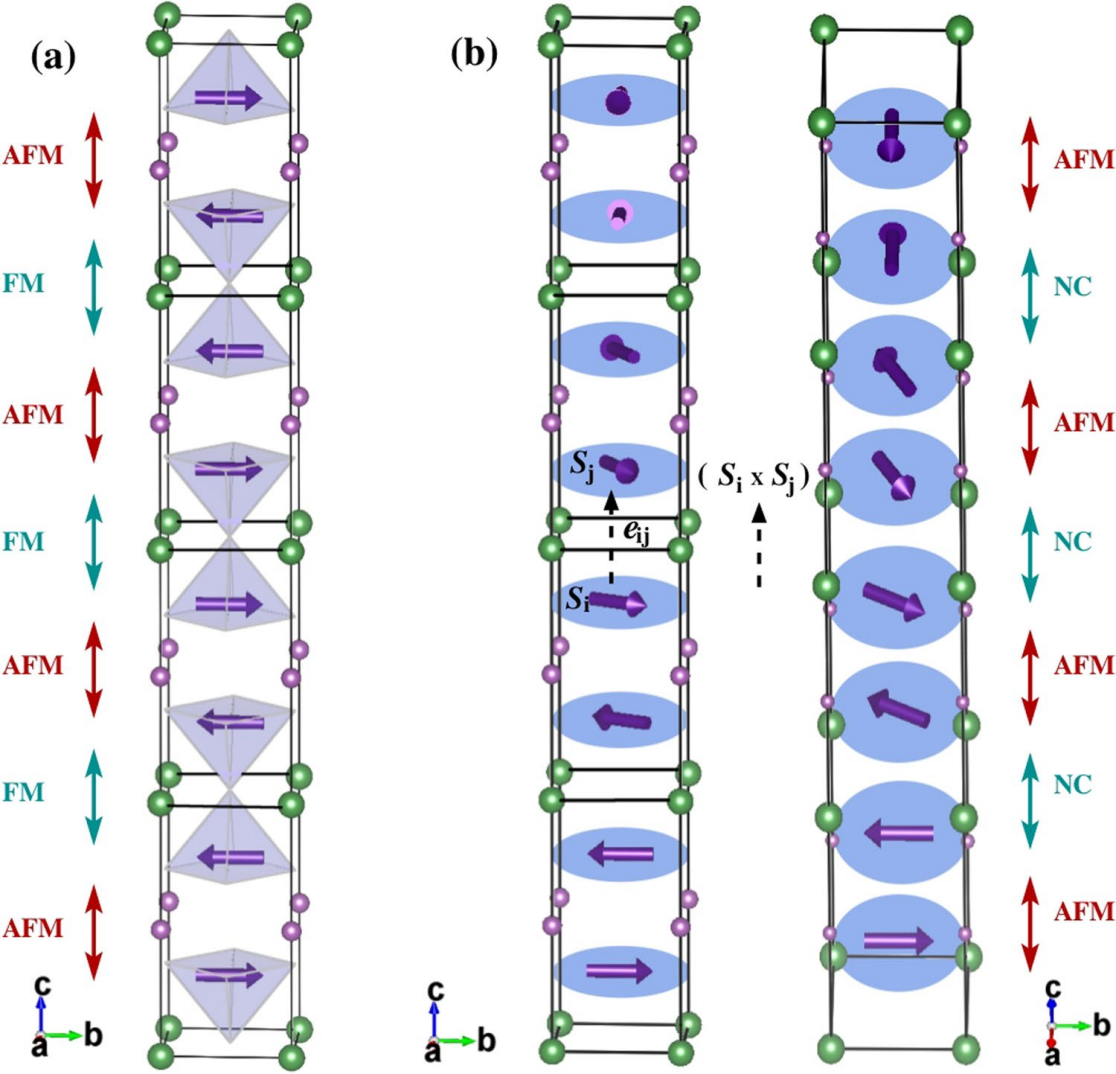
(**a**) Collinear magnetic order in CM phase (**b**) Helical magnetic spirals in ICM phase.

DFT calculations with SOC have been performed to compute the antisymmetric DM interaction parameter (**D**) as described in the literature^29^. The computed values for D_*x*_, D_*y*_ and D_*z*_ are found to be negligibly small (≈0.01 meV) to cause any significant electric polarisation in YBCFO. Such a tiny value of the interaction all but rules out DM interaction as a possible origin of magnetically induced ferroelectricity in YBCFO. Since the spin easy axis remains in the ab-plane, spins will prefer to remain in the plane when the system transits from CM to ICM phase. This implies that the nature of spiral state would be helical rather than cycloidal in the ICM phase. The proposed helical spiral state is shown in Fig. 2(b) where the non-colinearity appears within the ferromagnetically coupled bipyramids.

Another possible route to the existence of electric polarisation in the ICM phase is based on the spin-current model^23^ if the underlying magnetic spiral state is cycloidal in nature. According to this mechanism, the polarisation direction is given by **e**_*ij*_ × (**S**_*i*_ × **S**_*j*_), where **e**_*ij*_ is the vector connecting the i and j sites (see Fig. 2(b)) and spin indices are given by **S**_*i*_ and **S**_*j*_ respectively. However, if the spiral state is helical, as proposed here (shown in Fig. 2(b)), one can immediately see that the vector (**S**_*i*_ × **S**_*j*_) is along c direction and hence parallel to **e**_*ij*_. This will, of course, give zero electric polarisation. Therefore, in both cases the polarisation is going to be absent. We can, therefore, conclude with a reasonably degree of confidence that (i) the spins are oriented in the ab plane, (ii) spin spiral state in the ICM phase of YBCFO is helical rather than cycloidal, and (iii) there is no electric polarisation in the ICM phase of YBCFO. All these conclusions agree well with the single crystal measurements^14^.

### Effect of Sr doping on Magnetic Exchange Interaction

In order to see the effect of Sr doping on the various magnetic exchange interactions present in YBCFO, DFT calculations of YBa_1−*x*_Sr_*x*_CuFeO_5_ compound for *x* = 0, 0.25, 0.5, 0.75, 1 are performed. The calculated magnetic exchange interactions within LSDA + U for the doped compounds considering the CM magnetic structure as shown in Fig. 1(b) are listed in Table 2. The supercells of the doped compounds have been constructed by removing required concentration of Ba atoms and replacing them by Sr. For *x* = 0.5 we observe that alternate layers containing only Sr or Ba has lower energy than both layers having equal number of Sr and Ba ions. So we considered the former in the *x* = 0.5 structure. From Table 2 it is seen that *J*_*p*1_ increases with doping up to *x* = 0.5 and then decreases. One would therefore expect the PM to CM phase transition temperature (*T*_*N*1_) to also follow a similar trend. Along *c*, two types of NN interactions exist in the CM magnetic phase; one is the intra-bipyramidal ferromagnetic exchange between Fe and Cu and the other is inter-bipyramidal antiferromagnetic exchange between Fe-Fe and Cu-Cu pairs. We discuss below how various exchange interactions along c evolve with respect to doping.

#### NN-FM interaction

**Figure 3:**
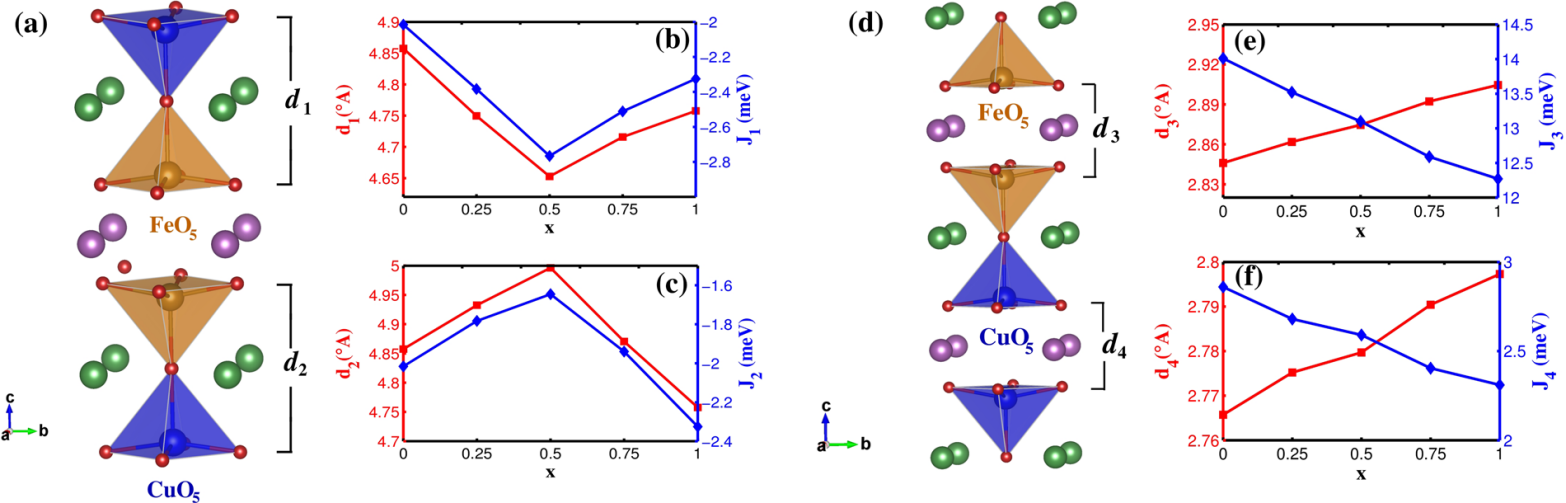
(**a**) d_1_(d_2_) marked in the crystal structure as the thickness of the Sr (Ba)-containing bipyramidal layers (**b**) Variation of d_1_ and *J*_1_ (**c**) d_2_ and *J*_2_ with doping (**d**) d_3_(d_4_) marked in the crystal structure as the inter bilayer distance separated by a layer of Y-ions (**e**) Variation of d_3_ and *J*_3_ (**f**) d_4_ and *J*_4_ with doping.

As Ba is replaced with Sr, having smaller ionic size, the thickness of bipyramidal layer starts to shrink from the corresponding value in the parent compound YBCFO. For example, at x = 0.25 the thickness of the bilayer containing Sr (d_1_) decreases, whereas the thickness of the bilayer not containing Sr (d_2_) increases (see Fig. 3). This change in the thickness is expected to affect the corresponding exchange interactions. From Fig. 3(b), ferromagnetic exchange interaction between Fe-Cu pairs (*J*_1_), corresponding to the bilayer containing Sr, increases. Whereas, the same for the bilayer that does not contain Sr (*J*_2_) decreases (Fig. 3(c)). This trend is followed for x = 0.5 as well where Sr and Ba segregate into different bilayers (see Fig. 3(b)).

For *x* > 0.5, when both the bilayers contain Sr ions, the dependence of (d_1_) and (d_2_) on doping is opposite to what is observed in *x* ≤ 0.5 case. So are the behaviour of *J*_1_ and *J*_2_. Note that at x = 1.0 (i.e. YSrCuFeO_5_) the FM exchange of both bilayers are equal and is slightly more than the corresponding exchange interactions for x = 0 (i.e. YBaCuFeO_5_). As it has been reported in the literature^14^ that the ICM phase is connected with the appearance of non-collinearity within the bilayers, the enhancement of FM exchange in YSrCuFeO_5_ (YSCFO) could imply that the CM phase would be more stable in this compound. The experimental reports on YSCFO compound^30,31^ indeed show that there is no CM to ICM phase transition.

#### NN-AFM interaction

In addition to its effect on the Fe-Cu ferromagnetic exchange interactions within the bipyramidal layers, Sr doping is also seen to influence the AFM exchange present between Cu-Cu and Fe-Fe ions in the adjacent bilayers (shown in Fig. 3(d–f)). The inter-bipyramidal distances (d_3_ and d_4_) are observed to increase with doping almost linearly. The corresponding AFM exchanges (J_3_ and J_4_) decrease monotonically with Sr doping. This effect is similar to what is observed in a recent experimental measurement in YBCFO with Fe/Cu chemical disorder^15^. The authors have reported that on increasing disorder the inter-bilayer distance increases whereas intra-bilayer distance decreases.

**Figure 4:**
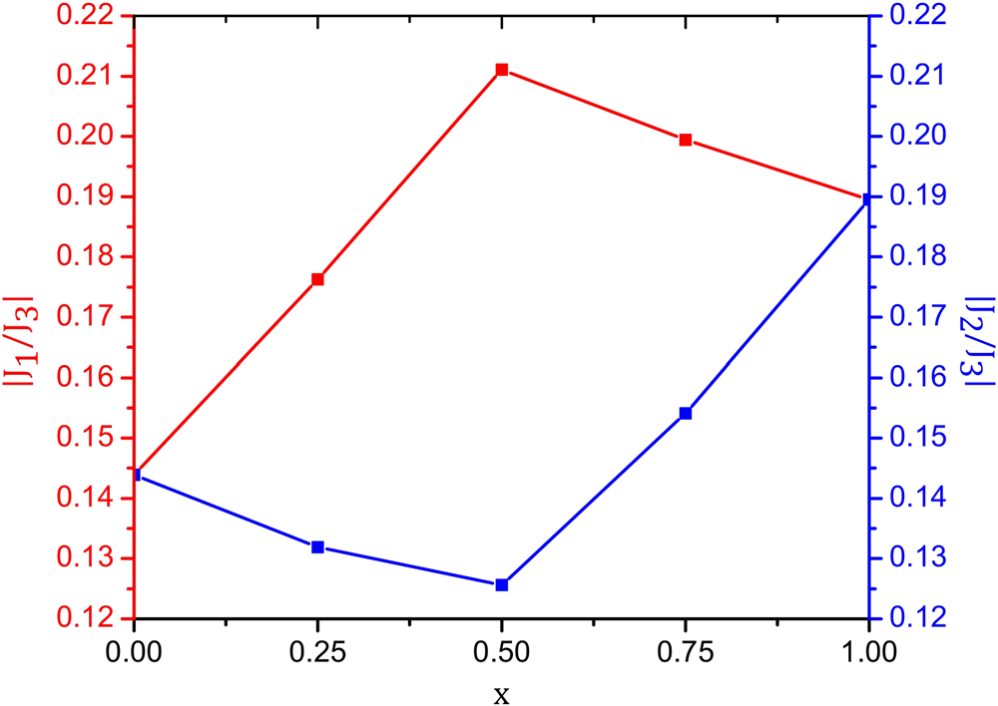
Variation of *J*_1_/*J*_3_ and *J*_2_/*J*_3_ with doping.

In Fig. 4 we have plotted the ratios *J*_1_/*J*_3_ and *J*_2_/*J*_3_ with doping, and we observe that these two ratios follow opposite trend with respect to the doping concentration. This indicates that the competition between these two interaction ratios may decide the magnetic phase diagram of these systems which is more complex than that due to Fe/Cu chemical disorder.

#### NNN interaction

**Figure 5:**
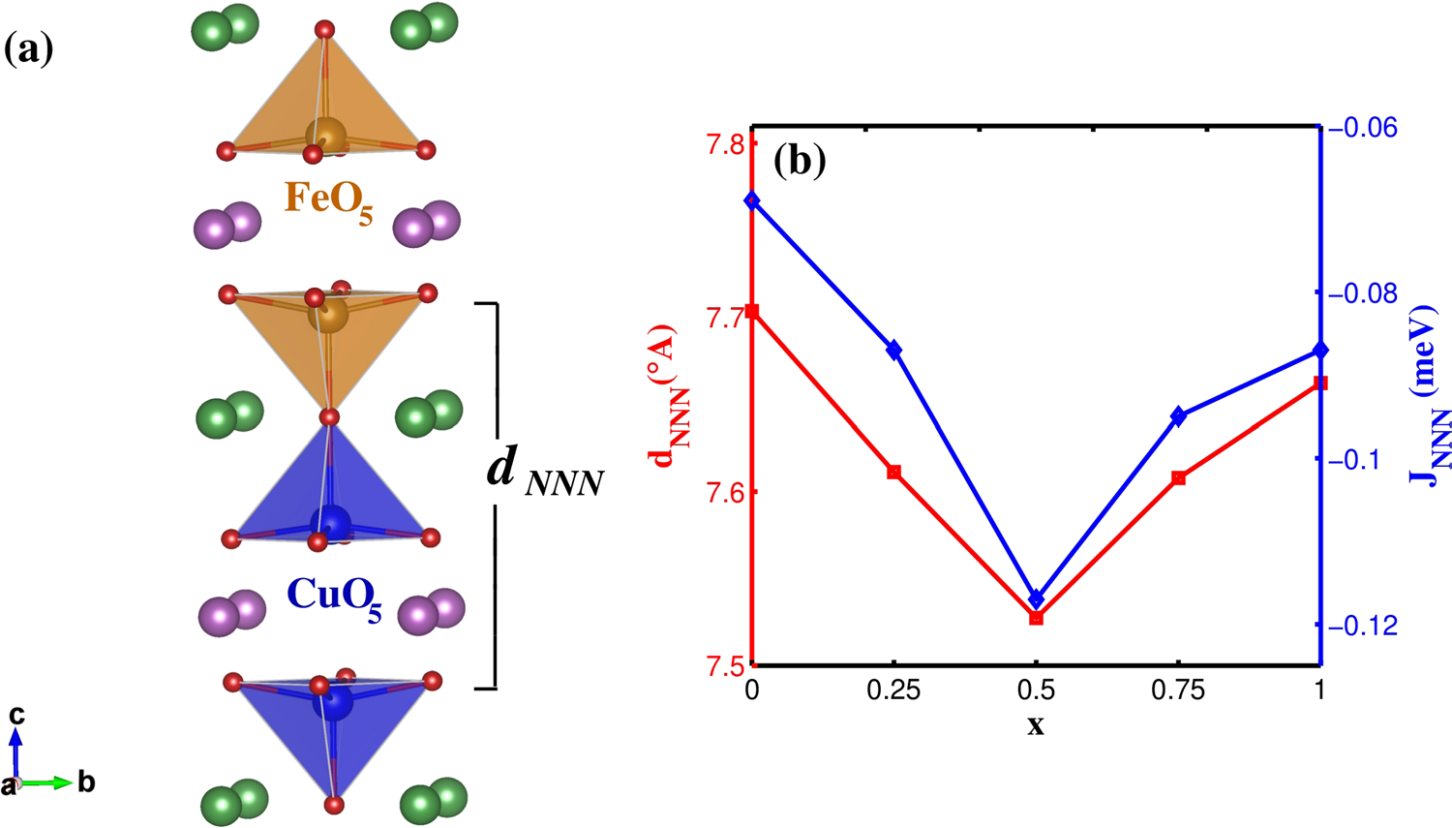
(**a**) d_*NNN*_ shown in the crystal structure (**b**) Variation of *J*_*NNN*_ with doping.

It is important to study the next nearest neighbour (NNN) exchange interaction with doping as it can play a very important role in stabilizing the ICM phase. In Fig. 5 we plot the calculated NNN exchange interaction strengths (*J*_*NNN*_) with respect to doping along with the corresponding NNN distance (d_*NNN*_). We observe that the interaction is ferromagnetic throughout the entire range of doping which is required to generate frustration in the CM phase. Though the strength by far is the smallest and it is seen to increase with Sr doping up to x = 0.5 and then decreases with higher doping. This result implies that the spiral state in the parent compound may get stabilized with doping at least up to x = 0.5 with Sr doping.

### Finite Temperature Calculations: QMC results

**Figure 6:**
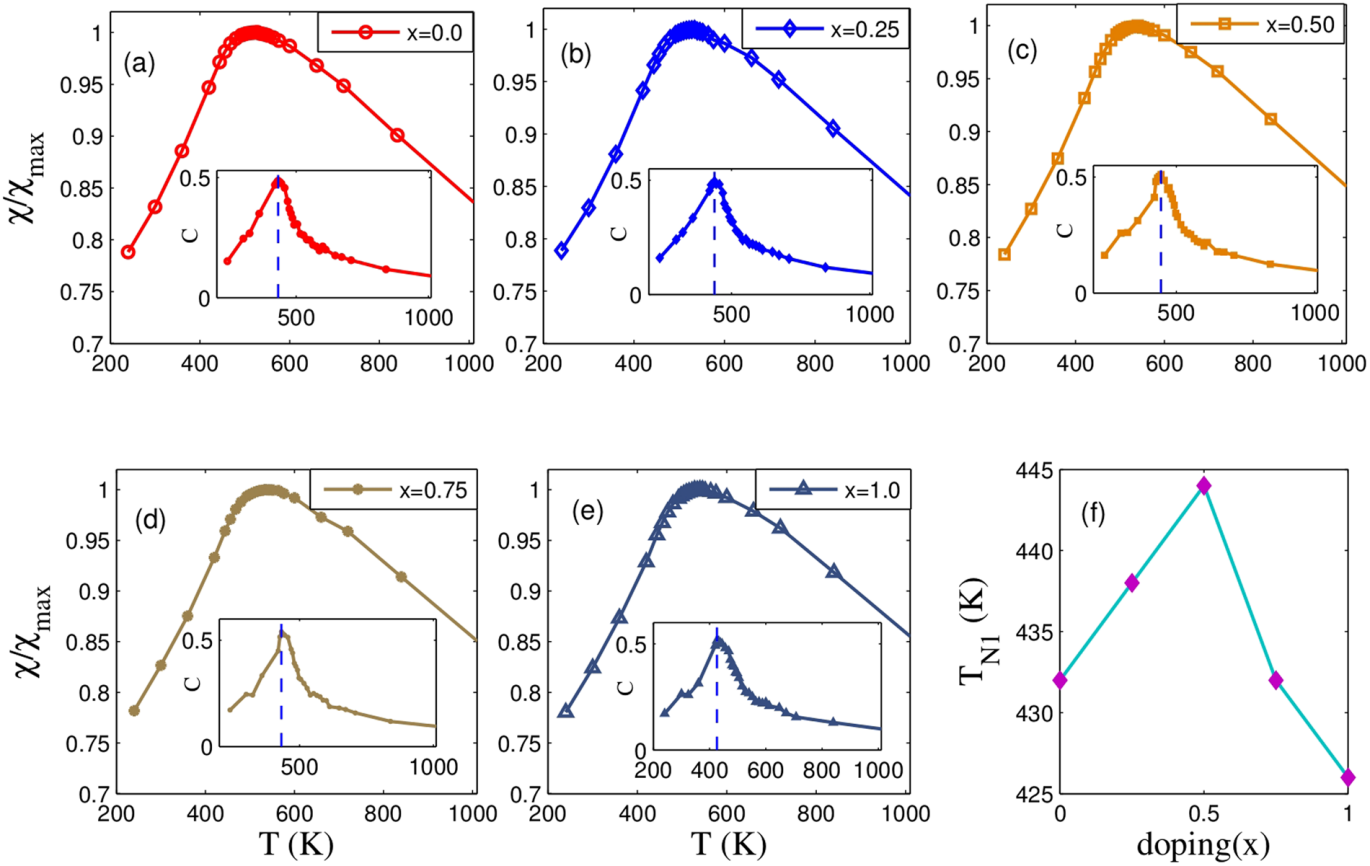
Magnetic susceptibility as a function of temperature at Sr doping (**a**) x = 0.00, (**b**) x = 0.25, (**c**) x = 0.50 (**d**) x = 0.75 (**e**) x = 1.00. Variation in the specific heat with temperature is shown in the inset, (**f**) Variation of T_*N*1_ (from the peak of the specific heat, dashed vertical line in (**a**–**e**) insets) with *x*.

Finally, we have performed QMC calculations with a Heisenberg spin Hamiltonian using the magnetic exchange interactions estimated from our DFT calculations as discussed above. In Fig. 6(a–e) we present the temperature-dependence of the magnetic susceptibility and specific heat (inset) at different Sr-doping. As usual, we identify the magnetic transition from the peak in the susceptibility and specific heat curves. In Fig. 6(f) we plot the variation of T_*N*1_ with doping. We observe that the transition temperature increases with doping up to *x* = 0.5 and reaches its maximum at 444 K. However, on further doping, the transition temperature starts to decrease. A comparison of calculated T_*N*1_s for the parent compounds (YBCFO and YSCFO) match very well with the previous experimental results^14,30^ on them. Experimental observations on PM to CM phase transition for YBCFO^14^ and YSCFO^30^ show that the T_*N*1_ is higher in case of YBCFO than that for YSCFO. This is also borne out quite well from our QMC results.

We note at this point that J_*NNN*_ has also been included in our QMC calculation but it fails to create enough frustration to stabilize the spiral phase.

## Conclusions

We have addressed the nature of spin spiral state and its correlation to the ferroelectric behaviour in the undoped YBCFO. The few experimental results that exist are not in agreement on this issue underlining the urgency of careful calculations. Using a detailed first-principles density functional theory, we clearly identify the magnetic spiral state to be a helical one where the spin moments rotate in the ab-planes without any out of plane component. The strength of the DM interaction is found to be exceedingly small, ruling out the role of this interaction in inducing ferroelectricity in YBCFO. We also show that a proposed mechanism, based on spin current model, cannot lead to finite electric polarisation in a helical spiral spin state. These findings are corroborated by the recent experimental observations^14^ on a high quality single crystal YBCFO sample, where the authors did not find any electric polarisation in the ICM phase and the spiral state is found to be helical rather than cycloidal.

In another related experiment^22^, it has been reported recently that the stability of the spiral state can be tuned with A site doping. We therefore performed a systematic study of magnetic phase transitions in Sr-doped YBCFO, for the entire range of doping, using DFT and QMC calculations. The structural changes due to doping is seen to affect various magnetic exchange interactions and the corresponding transition temperatures. Various NN and NNN exchange interactions between Fe-Cu, Fe-Fe and Cu-Cu spins are evaluated for the experimentally observed CM magnetic structure. We find a strong dependence of the exchange interactions on Sr doping. The Fe-Cu exchange (J_1_) along c direction, within the bipyramidal layer containing Sr, is ferromagnetic. It is found to increase with doping up to *x* ≤ 0.5. However, the magnetic exchange (J_2_), within a bipyramidal layer containing Ba, is seen to decrease. The reverse trend is observed for both J_1_ and J_2_ in the region *x* > 0.5. In addition, the inter-bipyramidal (separated by Y) AFM exchange couplings J_3_ and J_4_, between Cu-Cu and Fe-Fe pairs respectively, decrease monotonically with Sr doping.

Quite importantly, J_*NNN*_ is ferromagnetic; its value is found to increase with doping up to *x* = 0.5 though the strength is small compared to NN exchanges. It’s enhancement with doping, therefore, indicates that the spiral state in the parent compound (YBCFO) might be more stable with Sr doping at Ba sites, at least upto *x* = 0.5. From a QMC calculation on a spin Hamiltonian, whose spin exchange parameters are derived from DFT as described above, we observe that the PM to CM phase transition temperature T_*N*1_ increases with doping up to *x* = 0.5 and decreases beyond. These observations are quite consistent with the experimental data^22,30^ and show the way forward in doping-control of transition temperature in YBCFO.

## Methods

In order to study the electronic properties of the parent compound YBCFO and the Sr doped YBCFO (i.e. YBa_1−*x*_Sr_*x*_CuFeO_5_), we have employed the first-principles density functional theory (DFT)^32^ calculations at various levels of approximations such as local spin-density approximation (LSDA), LSDA + U and LSDA + U + SO. For our DFT calculations we have used the plane-wave and psuedopotential based method as implemented in the Vienna *ab-initio* Simulation Package (VASP)^33,34^ and projector augmented wave (PAW) potentials^35,36^. The wave functions were expanded in the plane-wave basis with a kinetic-energy cutoff of 600 eV. Reciprocal space integration was carried out with a k-mesh of 8 × 8 × 4.

Electron correlation effects beyond LSDA, important to properly describe the ground state of transition metal oxides, are incorporated using LSDA + U^37^ calculations where U is the on-site Coulomb correlation. We considered the value of U to be 5 eV for Fe and 8 eV for Cu. Corresponding Hund’s coupling strengths (J) were set to J_*Fe*_ = 1 eV and J_*Cu*_ = 0 for Fe and Cu respectively, as used in the previous literature^13^. Structural parameters were taken from experiments^22,30,31^ for the parent and all the doped structures (Table 1). Ionic positions for each structure are then optimized keeping the lattice constants at their respective experimental values.

SOC is incorporated in our calculations to determine the spin easy-axis or easy-plane anisotropy of parent YBCFO within LSDA + U + SO approximation. We have also performed non-collinear DFT calculations with SOC to estimate the antisymmetric DM interaction parameter.

Further, Quantum Monte-Carlo (QMC) calculations were carried out on a Heisenberg spin model (Eq. 1) using the loop algorithm^38^ of ALPS 2.1 package^39^, where *J*_*ij*_’s are magnetic exchange interactions between nearest neighbor (NN) and next nearest neighbor (NNN) bonds. **S**_*i*_ and **S**_*j*_ are the spin operators at site i and j respectively. We consider the spins associated with Fe^3+^ and Cu^2+^ ions are $\frac{5}{2}$ and $\frac{1}{2}$ respectively. The magnetic exchange interactions (NN and NNN) of the spin model were estimated (Table 2) from our DFT calculations to study the temperature-dependence of magnetic susceptibility and specific heat of these systems. We also study the doping-dependence of PM to CM transition temperature. These calculations were performed on a 16 × 16 × 32 supercell with periodic boundary condition in all three directions. To minimize statistical errors, we used 50000 sweeps for thermalization and 100000 measurement steps after thermalization in the temperature range from T = 840 K to 240 K.
